# A case study of monofilament line entanglement in a common bottlenose dolphin (*Tursiops truncatus*): entanglement, disentanglement, and subsequent death

**DOI:** 10.1186/s12917-020-02436-x

**Published:** 2020-06-30

**Authors:** Wendy Marks, Steve Burton, Elizabeth Stratton, Eric Zolman, George Biedenbach, Annie Page-Karjian

**Affiliations:** 1 Florida Atlantic University, Harbor Branch Oceanographic Institute, Fort Pierce, FL USA; 2 Earth Resources Technology Inc. contractor for NOAA Fisheries, Southeast Regional Office, St. Petersburg, FL USA; 3 National Marine Mammal Foundation, San Diego, CA USA; 4 Georgia Aquarium, Conservation Field Station, St. Augustine, FL USA

**Keywords:** Odontocete, Maxilla, Monofilament, Fisheries interaction

## Abstract

**Background:**

Free-ranging common bottlenose dolphins (*Tursiops truncatus*) can become entangled in fishing line and other marine debris. Infrequently, dolphins can be successfully disentangled, released back into the wild, and later examined postmortem to better understand the pathology and long-term effects of these entanglements.

**Case presentation:**

An entangled common bottlenose dolphin (*Tursiops truncatus*) calf was observed in the Indian River Lagoon, Florida, USA, with monofilament fishing line wrapped tightly around its maxilla. A multi-agency team successfully disentangled the dolphin for immediate release back into its natural habitat. A year after disentanglement, photos and observations indicated that the now independent calf showed a decline in body condition, characterized by grossly visible ribs and a prominent post-nuchal depression. More than 2 years post-disentanglement, the freshly dead carcass of this juvenile dolphin was recovered with extensive predation wounds. Despite the forestomach being ~ 50% full of ingesta (fish), the dolphin was emaciated. During postmortem examination, we collected and evaluated photographs and measurements of the maxillary damage resulting from the entanglement.

**Conclusion:**

The monofilament entanglement caused permanent, bilateral deformation of the maxillary dental arcade, including a 4.0–4.2 cm long, 0.5 cm deep linear groove where the entanglement eroded the lateral edges of the maxilla. There was no evidence of maxillary fracture and the dolphin survived for more than 2 years after disentanglement. External evidence of propeller scars and a fishing hook discovered embedded in the laryngeal mucosa at necropsy indicated repeated human interactions.

## Background

Fishing gear entanglements and evidence of previous entanglements are commonly observed in free-ranging and stranded common bottlenose dolphins (*Tursiops truncatus*) in Florida (FL), USA [1–4]. Such entanglements in cetaceans can impede movement and impair foraging abilities, leading to starvation, systemic infections, and debilitation from severe tissue damage, pain and distress, and in some cases, death [1, 3, 5].

The Indian River Lagoon (IRL) estuary system on the central east coast of FL stretches over 250 km, providing viable shallow water habitat for bottlenose dolphins. Local bottlenose dolphin populations demonstrate site fidelity [6, 7], making individuals relatively easy to re-sight. The shallow, soft substrate in several inshore areas along the IRL also allows for safe targeted capture and disentanglement efforts involving appropriately skilled biologists and veterinarians in cases where entanglements are deemed life threatening.

Reports of entanglements in bottlenose dolphins include a variety of anthropogenic materials, such as monofilament or multifilament fishing line and crab trap lines [3], as well as other items including human clothing, spearfishing gear, ring-shaped flying disc toys, box strapping, and other objects. It is rare, however, to encounter a case that can be followed from physically handling the dolphin at a disentanglement event to later recovering its carcass, enabling pathologic descriptions after an entanglement wound has healed. Here, we compare data collected on presentation during the disentanglement event to data collected at necropsy, demonstrating long-term damage from the entanglement involving maxillary bones, dentition and soft tissues of the rostrum.

## Case presentation

**Fig. 1:**
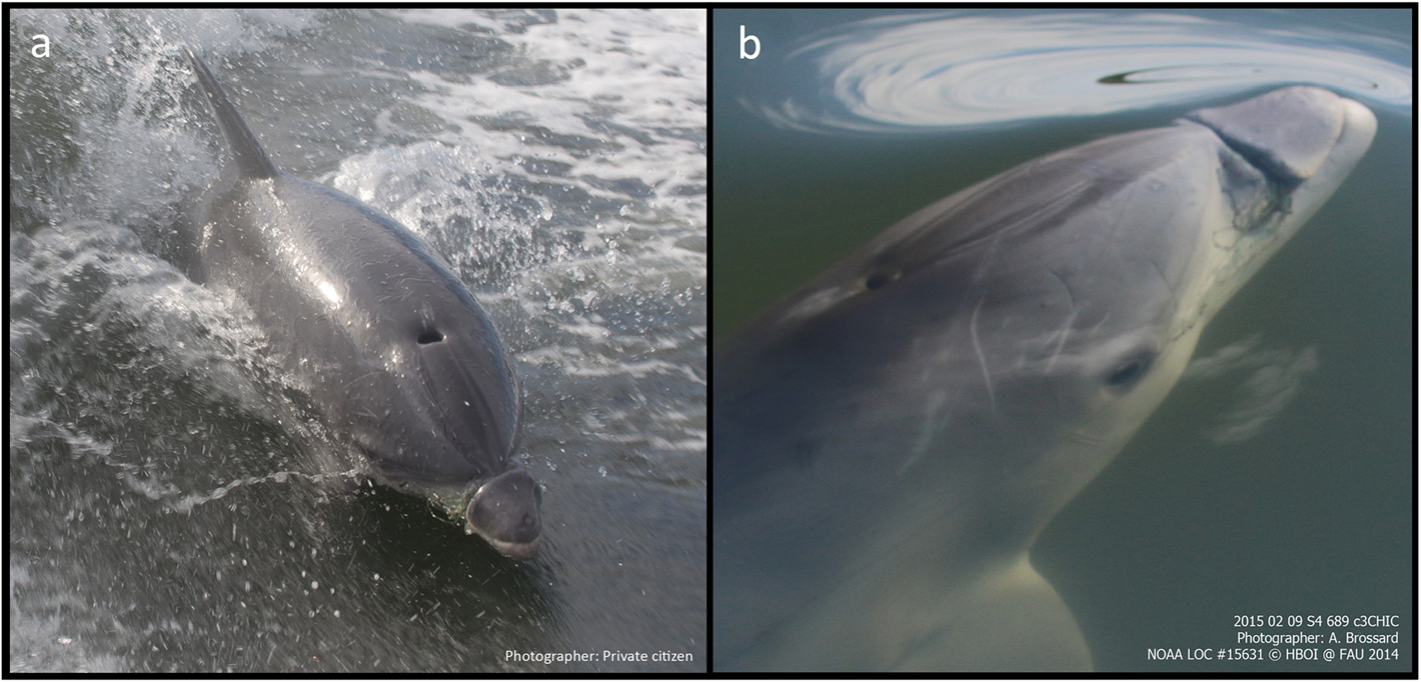
A free-ranging, male bottlenose dolphin (*Tursiops truncatus*) calf presented with maxillary entanglement in monofilament fishing line. **a** Initial photograph of the entanglement while the dolphin was wake riding on January 18, 2015 by a private citizen. **b** Follow-up photograph 22 days later (February 9, 2015) by a member of the FAU-HBOI Dolphin Photo Identification Team

**Fig. 2:**
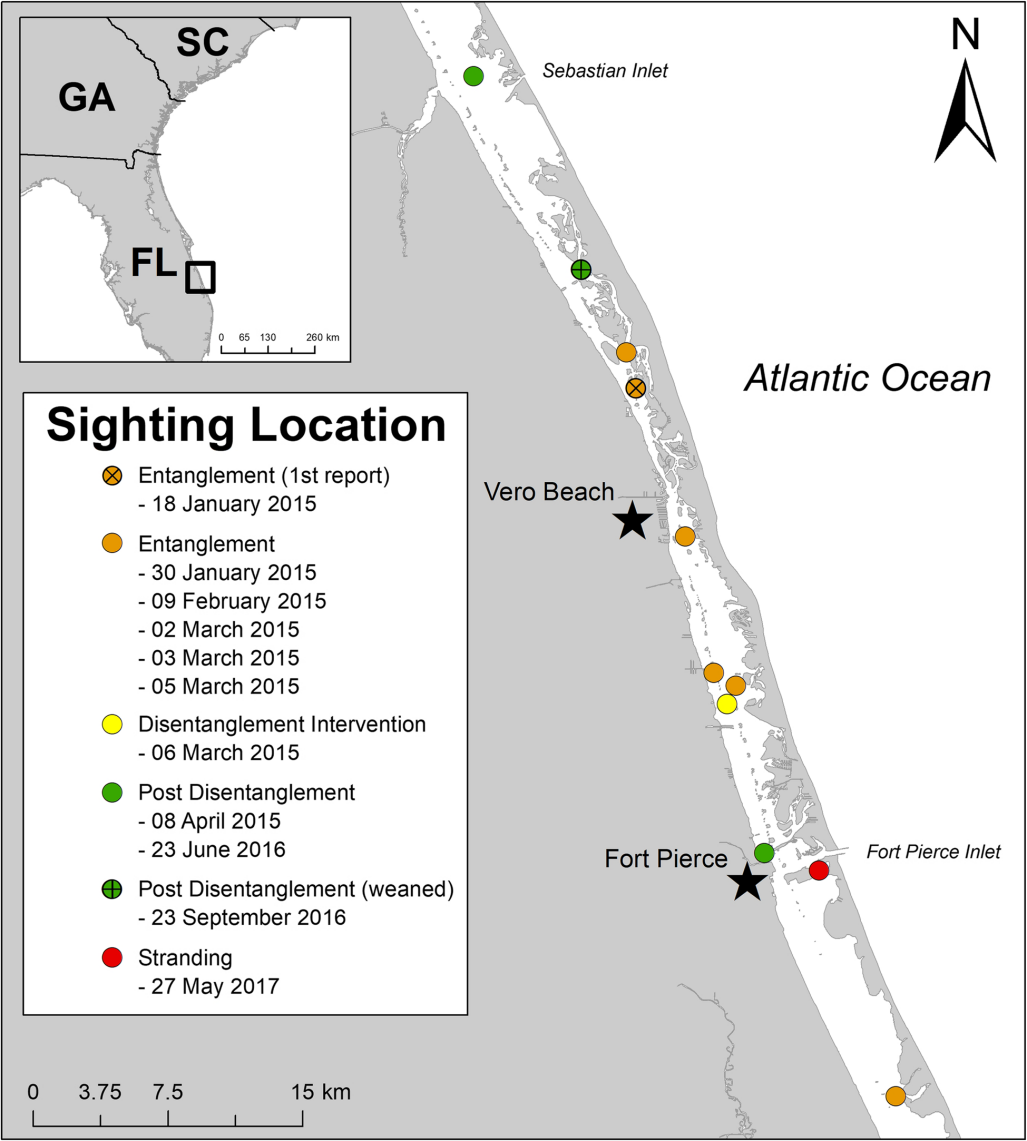
Map of the sighting locations for a common bottlenose dolphin (*Tursiops truncatus*) from the first report of the entanglement through the final stranding event. Map was created in ArcMap 10.6 (www.arcgis.com) (ESRI, Redlands, CA, USA)

A dependent male bottlenose dolphin calf was first reported with monofilament wrapped around his maxilla by a member of the public near Vero Beach, FL (27.704054°N, − 80.391446°W) on January 18, 2015. (Fig. 1a and Fig. 2). The Marine Mammal Rescue Team at Florida Atlantic University’s Harbor Branch Oceanographic Institute (FAU-HBOI) monitored this animal over several weeks to document changes to the entanglement and the animal’s body condition (Fig. 1b). Photos and observations indicated the calf to be of a normal to almost robust body condition initially, but showed a subtle decline in body condition, characterized by minor depressions caudal to the blowhole and over the scapulae. These data allowed for planning veterinary treatment during the disentanglement intervention.

**Fig. 3:**
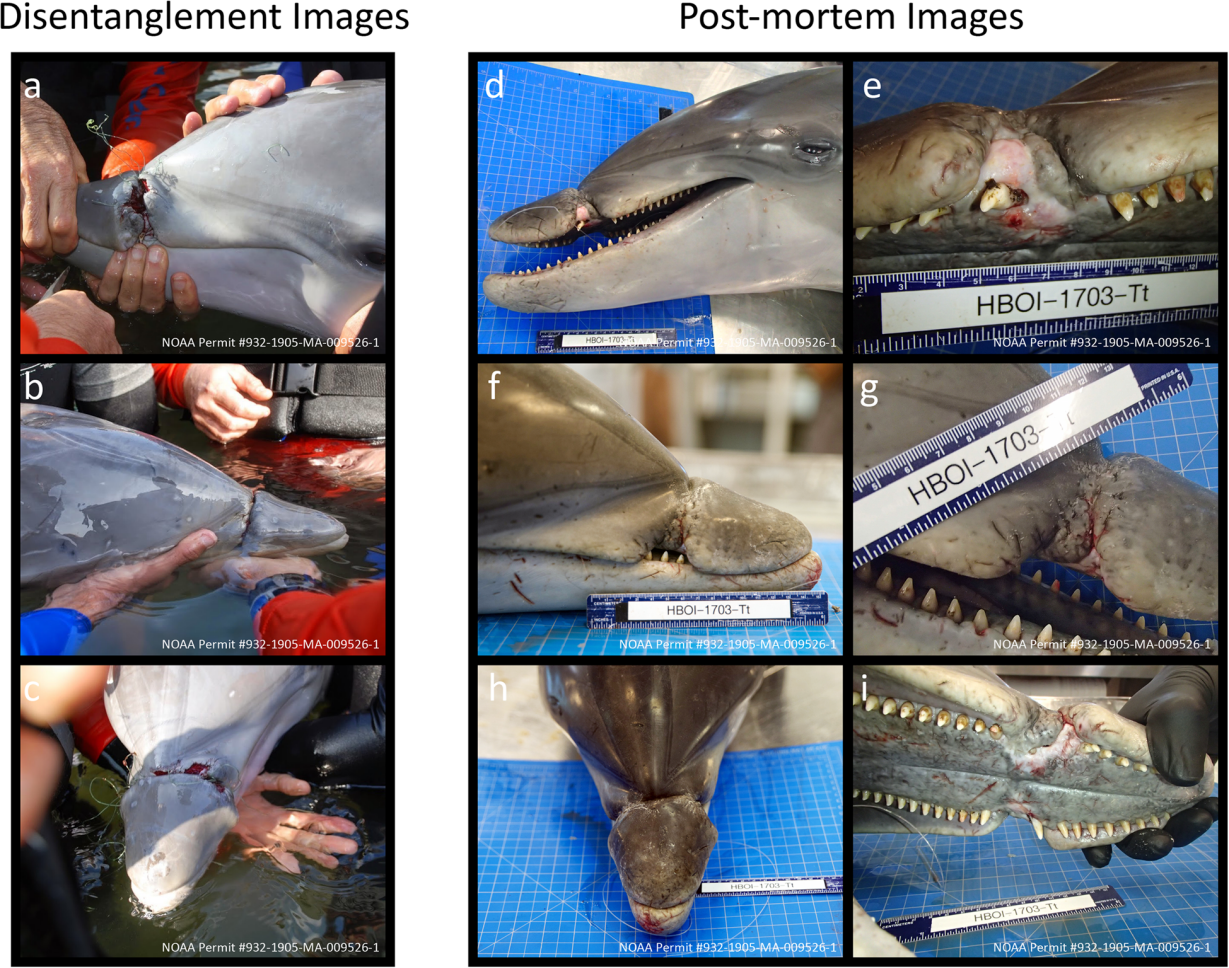
**a** Left lateral rostrum at disentanglement. **b** Right lateral rostrum at disentanglement. **c** Dorsal rostrum at disentanglement. **d** Left lateral rostrum postmortem. **e** Close-up of left lateral rostrum postmortem. **f** Right lateral rostrum postmortem. **g** Close-up of right lateral rostrum postmortem. **h** Dorsal rostrum postmortem. **i** View of hard palate and malocclusion postmortem

On March 6, 2015, the National Oceanic and Atmospheric Administration (NOAA) National Marine Fisheries Service (NMFS) authorized a successful multi-agency intervention near Fort Pierce, FL (27.545826°N, − 80,345,828°W) to catch the mother/calf pair in order to safely remove the entanglement (Fig. 3a-c) and assess the health of the calf. A large net encircled the mother/calf pair, and both were manually restrained for an in-water examination. After the 40–60 lb. test (approximate), green monofilament line with no hook was removed, veterinarians cleaned the calf’s rostrum wound and intramuscularly administered Ceftiofur (EXCEDE®, Zoetis Services LLC, Parsippany, NJ, USA), a long-acting broad-spectrum antibiotic, in the calf’s epaxial region using aseptic technique. Photographs were taken of the calf’s wound while both mother and calf were measured and blood samples were collected. The three attending veterinarians concurred that the calf’s health status did not warrant admission into rehabilitation, and that the animal was best left to heal in its natural habitat. The mother was fitted with a VHF radio tag to facilitate follow-up monitoring of the calf and they were successfully released in situ.

The calf was observed with his mother on several occasions over the next year by the FAU-HBOI Marine Mammal Rescue and Dolphin Photo Identification Teams. In fall of 2015, parallel full-thickness linear and wedge-shaped defects were observed and attributed to propeller strike [8]. In the summer of 2016, the mother was seen with a new calf. On September 23, 2016, the formerly entangled, juvenile dolphin was first re-sighted alone and presumed to be weaned from his mother. At that time, he was judged to be emaciated based on appearance of a post-cranial dip (“peanut head”) and ribs that were visible upon surfacing.

**Fig. 4:**
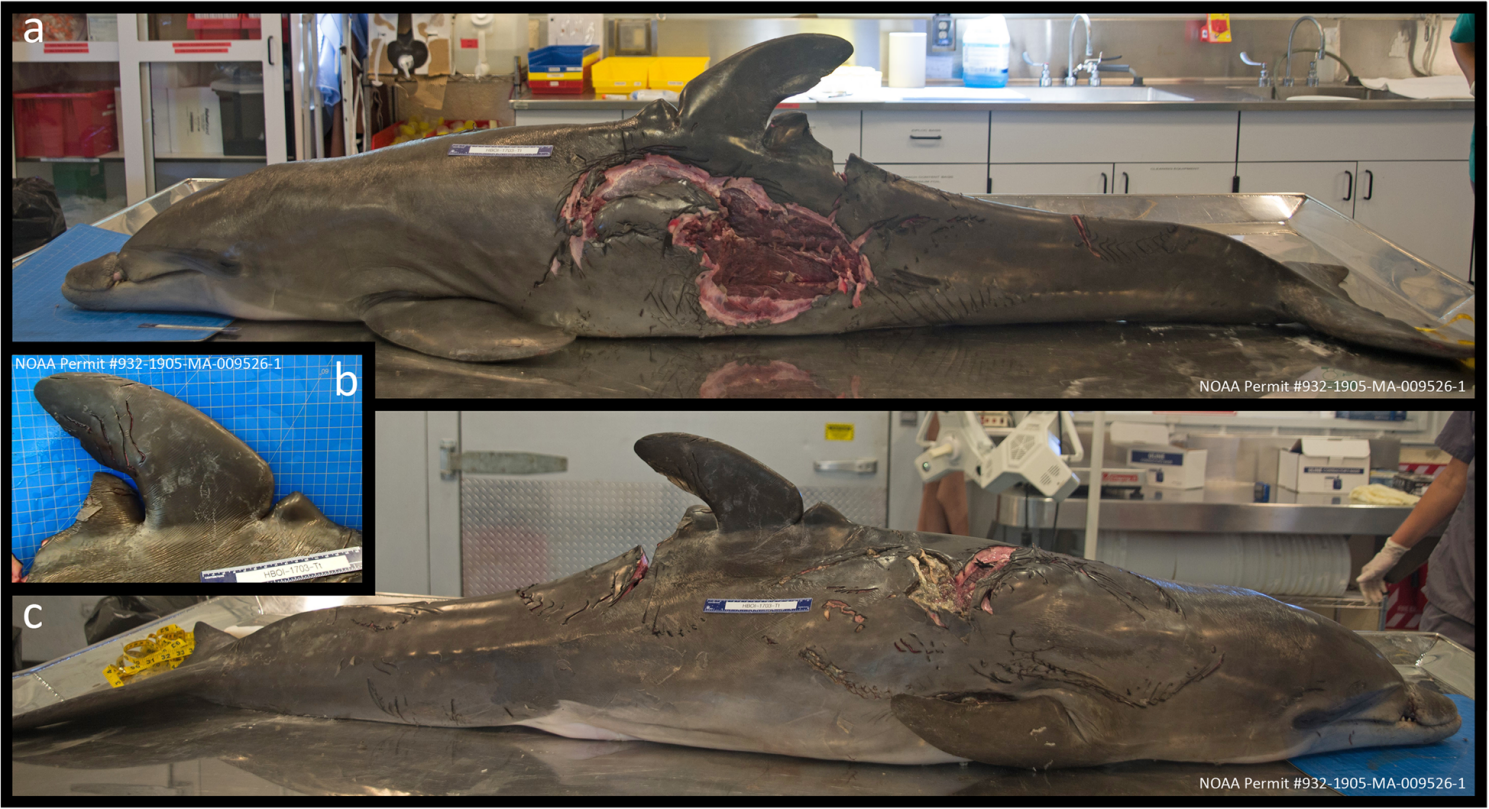
External body images prior to necropsy demonstrating multiple shark bites, and healed propeller wounds to the dorsal fin. **a** Left lateral full body view. **b** Right lateral view of dorsal fin highlighting healed vessel strike scarring. **c** Right lateral full body view

On May 27, 2017, the dolphin’s carcass was recovered floating along a seawall in the IRL, along the north entrance to Faber Cove, Causeway Island, Fort Pierce FL (27.462394°N, − 80.299675°W) with several shark bites along the lateral and ventral sides of his body, the largest extending 29 cm × 21 cm along the left lateral side and invading deep into the musculature (Fig. 4a, c). There were two 6 cm deep, healed wounds perpendicular to the dolphin’s dorsal fin, presumably from a previous boat propeller strike (Fig. 4b). Fishing line extended from the dolphin’s mouth. Postmortem photographs and measurements were taken of the rostrum at the entanglement site (Fig. 3d-i), as well as full body photos and close-up photos of any other external anomalies. After these photos were taken, a complete gross necropsy was conducted at the FAU-HBOI necropsy laboratory. No remaining foreign material or line was evident in the maxillary lesion at the time of the necropsy. Post necropsy, the soft tissue was cleaned from the skull, and the skull was placed into a hydrogen peroxide bath to remove all traces of soft tissue, thereby enabling direct visual examination of the skull.

**Fig. 5:**
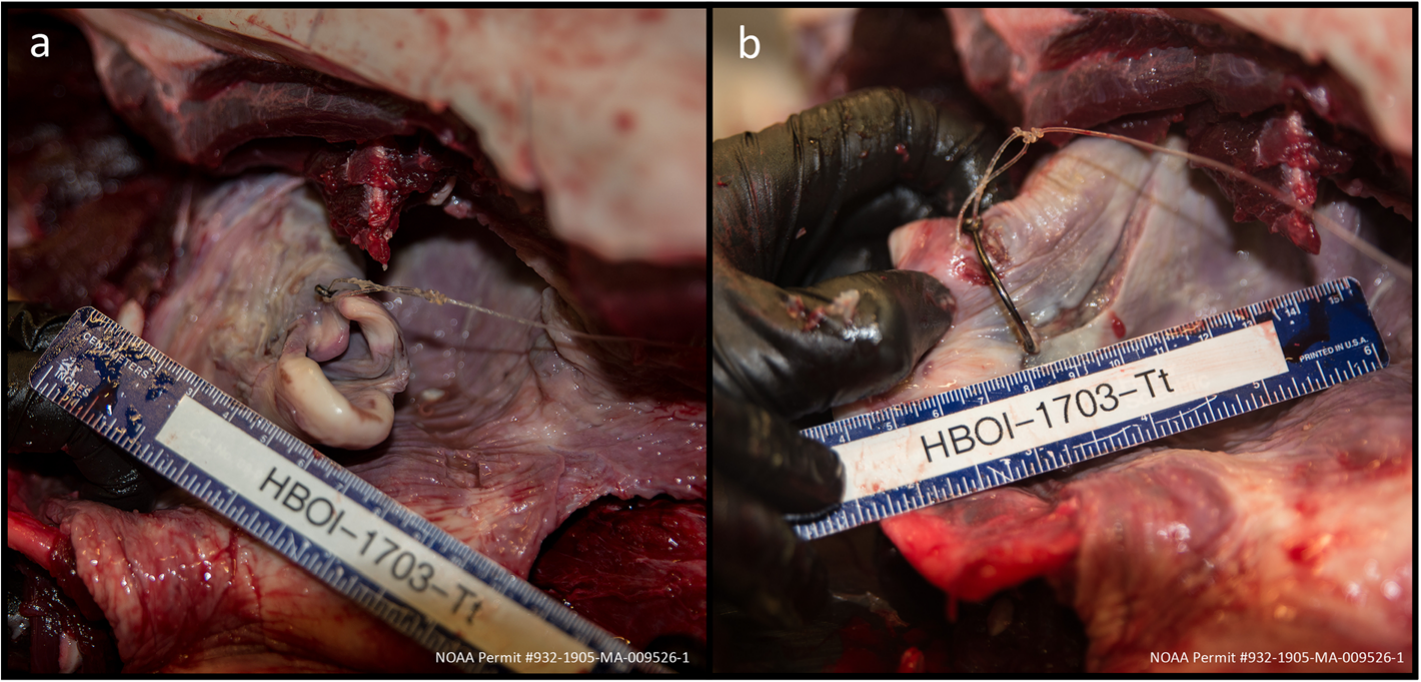
**a** Cranial view of the hook embedded into the dolphin’s laryngeal opening (goosebeak), with fishing line extended cranially. **b** Hook insertion on the left lateral side of the goosebeak with a linear, partially healed esophageal mucosal laceration demonstrating the path of the hook through soft tissues, and the eye of the hook causing tissue erosion dorsally

Internal gross examination revealed that the extant 40-lb. test monofilament fishing line leader extending from the mouth was anchored to a 6/0 circle hook embedded ~ 2 cm caudal to the left lateral side of the laryngeal (goosebeak) opening (Fig. 5a). Multiple lesions were present within the esophagus in proximity to the hook that were histologically composed of organized granulation tissue (Fig. 5b), and a partially digested fish was located in the esophagus just caudal to the hook. The forestomach was approximately 50% full of ingesta, and contained fish bones, 15 fish otoliths, a sheepshead (*Archosargus probatocephalus*) jaw with teeth, and a fish gizzard, with no mucosal ulcerations or parasites observed. The stomach, pylorus, and duodenal ampullae were grossly normal and free of digesta or parasites. Although the presence of partially digested food demonstrated that the dolphin had been eating, emaciation was evident by a prominent post-nuchal depression, externally visible ribs and vertebral processes, thin blubber layer (0.9 cm - 1.0 cm), and atrophied epaxial muscles (Fig. 4a, c). Histological examination of cutaneous wounds indicated that the extensive shark bite wounds and accompanying hemorrhage occurred antemortem. The esophageal lesion surrounding the embedded hook was characterized by severe, chronic, focal submucosal granulation tissue with re-epithelialization and prominent lymphoid follicles. The embedded hook may have partially impeded breathing and/or eating, and likely contributed to this animal’s failure to thrive and to his ultimate demise. Bilateral verminous, pyogranulomatous pneumonia was observed in the lungs, associated with nematodes (80 μm diameter with a thin, smooth cuticle, likely *Halocercus* spp.) and accompanied by pulmonary smooth muscle hyperplasia and constriction [9]. There was also moderate, acute, diffuse, bilateral pulmonary edema. Multiple lymph nodes (pulmonary, mesenteric, genital) contained moderate numbers of resorbed eosinophils, which may be indicative of systemic eosinophilia. Lymphoid depletion was not observed, and whole blood and blowhole swab samples tested negative for dolphin morbillivirus via reverse-transcriptase PCR (Marine Ecosystem Health Diagnostics & Surveillance Laboratory, University of California, Davis). No further diagnostics were performed.

**Fig. 6:**
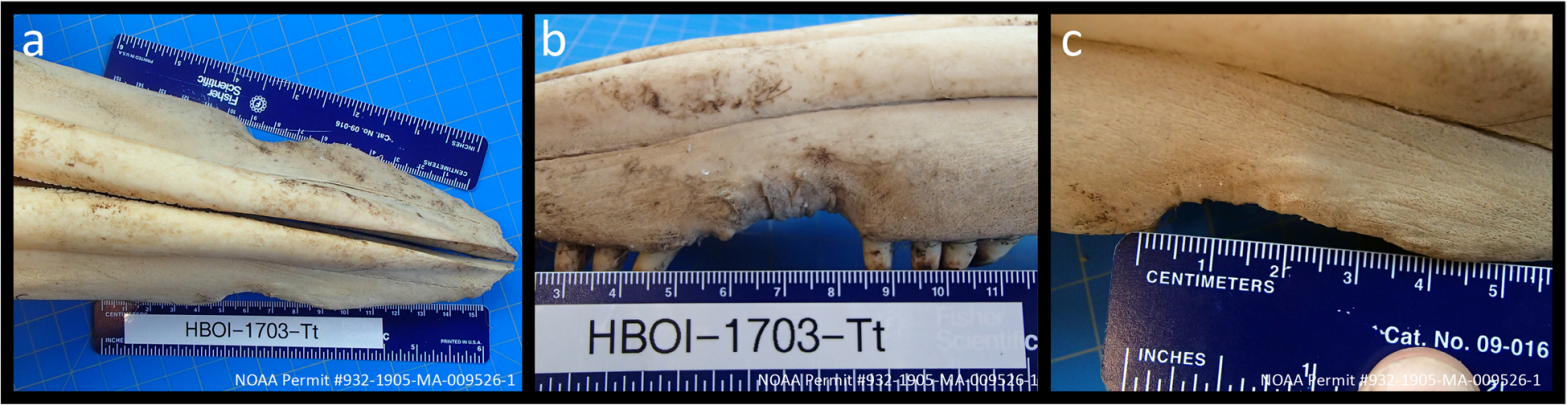
There is a 4.0 cm long by 0.5 cm deep defect on the left lateral edge of the maxilla. Visible within the larger defect are 4 smaller grooves, each about 1 mm deep. There is also a defect on the right lateral maxillary edge, 4.2 cm long by 0.5 cm deep, with 5 smaller individual grooves that are each less than 1 mm long. **a** Dorsal view of maxilla shows the bilateral bony erosion of the maxilla. **b** Left lateral maxillary defect. **c** Right lateral maxillary defect

The monofilament entanglement around the maxilla in 2015 led to maxillary malocclusion, including a gap between the upper 8th and 9th teeth on the left side (Fig. 3d-i). On the right side, the 8th tooth is deflected medially and buried in thickened scar tissue with only a small piece of the tooth visible. The upper left 10th and 11th teeth and the upper right 9th and 10th teeth are missing. The two teeth caudal to the missing teeth are shifted slightly medially with mild bilateral dental crowding. The left maxilla has a 4.0 cm long by 0.5 cm deep defect on the lateral edge with 1 mm deep individual grooves visible within the larger defect (Fig. 6a-c). The right maxilla has a 4.2 cm long by 0.5 cm deep defect on its lateral edge. The smaller individual grooves are < 1 mm deep but are still visible grossly. No fractures or calcifications from bone remodeling were observed upon close visual inspection.

## Discussion and conclusions

Recreational fishing spots often coincide with bottlenose dolphin feeding habitats due to a shared targeting of the same fish species. Commonly observed interactions between dolphins and recreational fishermen include dolphin depredation of bait or captured fish, illegal feeding of dolphins, and dolphin encounters during release of undersized or non-targeted fish [10, 11]. A study of bottlenose dolphins in Sarasota Bay, FL found that dolphins known to interact with fishing gear were more likely to be found within 50 m of fishing lines [11]. Educating fishermen about the importance of reeling in their lines when dolphins are present and proper disposal of fishing line can help reduce the risk of these interactions. Less lost fishing gear and fewer injured or dead marine wildlife benefits everyone. In this case, the ~ 1.5-month monofilament entanglement did not cause bone fracture but did lead to bone deformity and maxillary malocclusion. While the quick response to disentangle this dolphin may have initially saved the dolphin’s life, prevention of learned fishery interaction behaviors could have prolonged his life beyond the 2+ years that were gained.

## Data Availability

Not applicable.

## Abbreviations

FL: Florida
IRL: Indian River Lagoon
FAU-HBOI: Florida Atlantic University’s Harbor Branch Oceanographic Institute
NOAA: National Oceanographic and Atmospheric Administration
NMFS: National Marine Fisheries Service
